
JOURNAL INFORMATION
==============================
NLM Title Abbreviation: Biospektrum (Heidelb)
Journal ID: Biospektrum (Heidelb)
NLM Journal ID: 9615685

Biospektrum

ISSN: 0947-0867
EISSN: 1868-6249

ARTICLE INFORMATION
==============================
PMCID: PMC9942070
PMID: 36845577
DOI: 10.1007/s12268-023-1881-3
Article ID: 1881
Article version: 1
Subjects: Wissenschaft · Special: Genomics & Epigenetics

Lichtgesteuerte Translation von mRNA in Eukaryoten

Dittmar Maria
Weissenböck Florian Peter
Rentmeister Andrea a.rentmeister@uni-muenster.de

grid.5949.1 0000 0001 2172 9288 Institut für Biochemie, Westfälische Wilhelms-Universität Münster, Corrensstraße 36, D-48149 Münster, Deutschland
Electronic publication date: 2023 Feb 21
Print publication date: 2023
Volume: 29
Issue: 1
First page: 31
Last page: 34
Copyright: © Die Autorinnen und Autoren 2023
License: Open Access: Dieser Artikel wird unter der Creative Commons Namensnennung 4.0 International Lizenz veröffentlicht, welche die Nutzung, Vervielfältigung, Bearbeitung, Verbreitung und Wiedergabe in jeglichem Medium und Format erlaubt, sofern Sie den/die ursprünglichen Autor(en) und die Quelle ordnungsgemäß nennen, einen Link zur Creative Commons Lizenz beifügen und angeben, ob Änderungen vorgenommen wurden. Die in diesem Artikel enthaltenen Bilder und sonstiges Drittmaterial unterliegen ebenfalls der genannten Creative Commons Lizenz, sofern sich aus der Abbildungslegende nichts anderes ergibt. Sofern das betreffende Material nicht unter der genannten Creative Commons Lizenz steht und die betreffende Handlung nicht nach gesetzlichen Vorschriften erlaubt ist, ist für die oben aufgeführten Weiterverwendungen des Materials die Einwilligung des jeweiligen Rechteinhabers einzuholen. Weitere Details zur Lizenz entnehmen Sie bitte der Lizenzinformation auf http://creativecommons.org/licenses/by/4.0/deed.de.
License URL: https://creativecommons.org/licenses/by/4.0/

issue-copyright-statement: © Der/die Autor(en), exklusiv lizenziert an Springer-Verlag GmbH Deutschland, ein Teil von Springer Nature 2023

==============================
Messenger RNA (mRNA) shows great potential for medical applications, as recently demonstrated by the mRNA-based vaccines against the coronavirus. In addition, it has long been used for ectopic gene expression in cells and model organisms. While numerous methodologies are available for controlling gene expression at the level of transcription, approaches to control translation are scarce. Here we review strategies for direct light-mediated activation of mRNA translation via photocleavable groups and their potential to achieve spatial and temporal control of protein production.

■ Boten-RNA (mRNA) hat großes Potenzial für medizinische Anwendungen, wie die mRNA-basierten Vakzine von Moderna und BioNTech/Pfizer kürzlich eindrucksvoll gezeigt haben [1]. Auch in der Krebsimmun- und der Proteinersatztherapie setzt man große Hoffnung auf mRNA. Seit langem wird mRNA außerdem in der Grundlagenforschung für die ektopische Genexpression in Zellen und Modellorganismen eingesetzt. Allerdings fehlen Ansätze zur Kontrolle der Translation exogener mRNA, während zur Kontrolle der Genexpression auf Ebene der Transkription zahlreiche Methoden zur Verfügung stehen. Wir fassen hier Strategien zur lichtvermittelten Aktivierung der Translation zusammen. Diese basieren auf Photoschutzgruppen und haben das Potenzial, die Translation von exogener mRNA räumlich und zeitlich präzise zu aktivieren

## Eukaryotische Translation

Die Proteinbiosynthese in Eukaryoten ist ein komplexer Prozess, der mit der Translationsinitiation beginnt (**Abb. 1A**). Zwei Charakteristika der eukaryotischen mRNA – die 5'-Kappe und der 3'-Poly(A)-Schwanz (**Abb. 1B**) – spielen dabei eine wichtige Rolle. Zunächst bindet der eukaryotische Translationsinitiationsfaktor 4F (eIF4F), ein Komplex aus eIF4E, eIF4A und eIF4G, an die mRNA. Dabei ist entscheidend, dass eIF4E die 5'-Kappe erkennt und direkt bindet (**Abb. 1C**). Andere Faktoren und Poly(A)-Bindeproteine (PABP) kommen hinzu und bewirken die Zirkularisierung durch Wechselwirkungen zwischen den eIFs am 5'-Ende und den PABP am 3'-Ende der mRNA. Dieser Schritt führt zur Rekrutierung der kleinen ribosomalen Untereinheit (40S), die die folgenden Initiationsphasen einleitet. Final kommt es zur Assemblierung des Ribosoms (80S) und die Translation beginnt [4].

## Strategien zur lichtgesteuerten mRNA-Aktivierung

Die Genexpression posttranskriptional in Raum und Zeit zu kontrollieren, ist sowohl für therapeutische Anwendungen

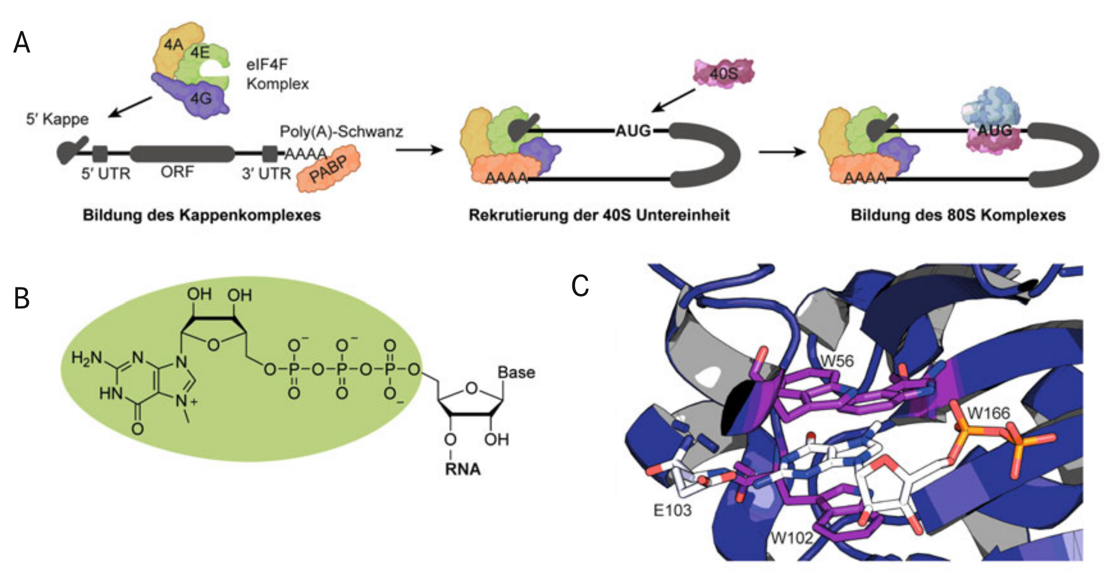

◀ **Abb. 1:** Die mRNA-5'-Kappe, ein Charakteristikum eukaryotischer mRNA, leitet die kappenabhängige Translation ein. **A**, Ausbildung des Kappenkomplexes. **B**, exemplarische Kappe einer eukaryotischen mRNA. **C**, Kristallstruktur von eIF4E mit 5'-Kappe und hervorgehobenen wichtigen interagierenden Aminosäuren.

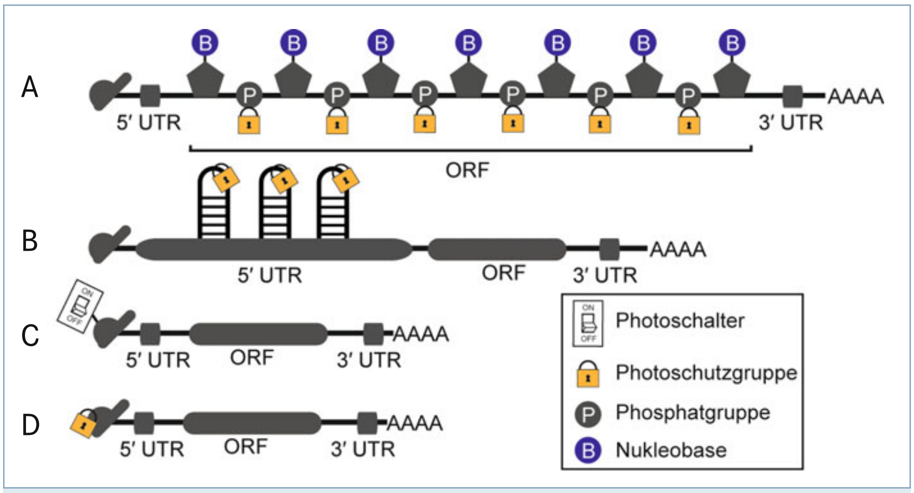

▲ **Abb. 2:** Strategien zur lichtinduzierten Translationskontrolle auf mRNA-Basis. **A**, Photoschützung des Phosphatrückgrats der mRNA. **B**, mRNA mit drei Erkennungssequenzen zur chemo-enzymatischen Photoschützung. **C**, Photoschalter an der 5'-Kappe der mRNA. **D**, photogeschützte mRNA-Kappe (Flash-Cap). UTR: untranslatierte Region; ORF: *open reading frame*.

als auch für Fragestellungen in der Grundlagenforschung, besonders in der Entwicklungsbiologie, von großem Interesse. Möchte man unabhängig von Promotoren und Enhancern sein, benötigt man hierfür einen Induktor (*trigger*), der jederzeit präzise eingesetzt werden kann und weder cytotoxisch ist, noch unerwünschte Effekte im biologischen System bewirkt. Licht hat sich für die meisten Zelltypen als besonders geeignet erwiesen und kann sehr gezielt appliziert werden, wie zahlreiche optogenetische Anwendungen zeigen [5, 6].

Seit Jahrzehnten kennt man in der Chemie funktionelle Gruppen, die sich mithilfe von Licht abspalten (Photoschutzgruppen) oder isomerisieren (Photoschalter) lassen. Durch das Einbringen solcher photoresponsiver Gruppen in Biomoleküle können biologische Prozesse mit Licht kontrolliert werden, wie am Beispiel photogeschützer ATPs zur Untersuchung ATP-abhängiger extra- und intrazellulärer Prozesse gezeigt wurde [7].

Für die posttranskriptionale Regulation der Genexpression wurden in den letzten Jahren vor allem kurze, synthetisch hergestellte Oligonukleotide mit Photoschutzgruppen versehen. Die Blockierung der Watson-Crick-Basenpaarung photogeschützter microRNAs, Morpholinos und Antisense-Oligonukleotide in Kombination mit der lichtinduzierten Aufhebung ermöglichten die Kontrolle der Translation endogener mRNAs mit Licht. Zu berücksichtigen sind allerdings mögliche Off-Target-Effekte und die indirekte Funktionsweise, da sie einem weiteren biologischen Prozess unterliegen, wie dem RNase-H- oder dem *RNA induced silencing complex*(RISC)-mRNA-Abbau [8].

Methoden, die nicht auf der Interaktion eines biologisch relevanten Moleküls mit der

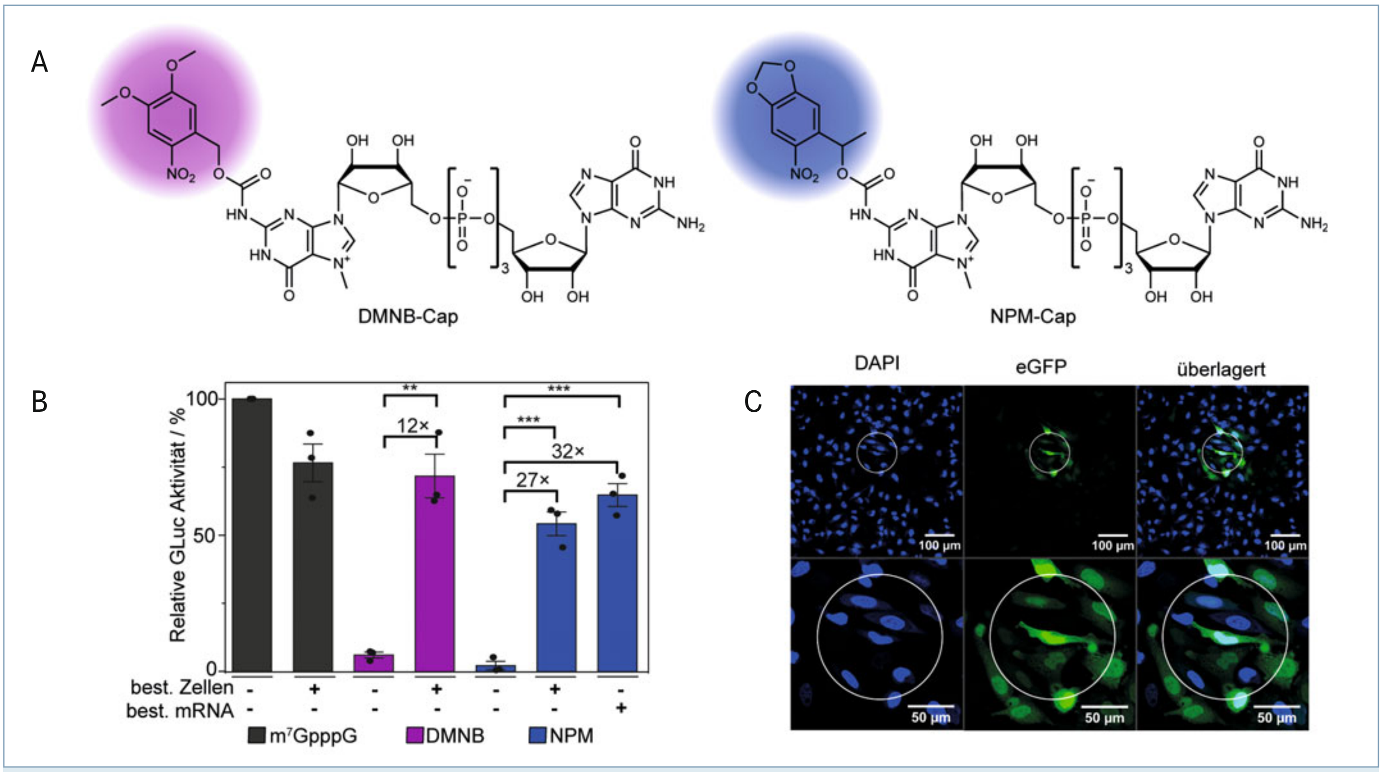

▲ **Abb. 3:** Lichtaktivierte Translation mithilfe von Flash-Caps. **A**, chemische Struktur der Flash-Caps. **B**, Vergleich der relativen Luciferaseaktivität nach Translation von GLuc-mRNAs mit unterschiedlichen 5'-Kappen in transfizierten HeLa-Zellen. **C**, lokale Bestrahlung von mit Flash-Cap-eGFP-mRNA transfizierten HeLa-Zellen mit einem konfokalen Laser-Scanning-Mikroskop im eingekreisten Bereich. Zellkerne wurden mit DAPI eingefärbt (blau). Aus [12], CC BY 4.0.

mRNA beruhen, sondern die Translation der mRNA durch eine direkte Modifikation beeinflussen, sind hingegen noch selten. Aktuell kann man vier Strategien zur lichtgesteuerten Aktivierung der mRNA-Translation unterscheiden (**Abb. 2**).

Die Gruppe von Prof. Okamoto (Riken Center for Brain Science, Wakō) hat bereits im Jahr 2001 mRNA chemisch modifiziert (**Abb. 2A**). Hierfür wurde die durch *in vitro*-Transkription (IVT) hergestellte mRNA mit 6-Bromo-4-diazomethyl-7-hydroxycumarin umgesetzt. Dadurch wurde das Phosphatrückgrat der mRNA mit den Photoschutzgruppen modifiziert, wobei ungefähr 30 Photoschutzgruppen pro 1.000 Nukleotide angebracht wurden (**Abb. 2A**). Die photogeschützte mRNA zeigte, wie gewünscht, in Zebrafischen keine Translation. Allerdings konnte auch nach Bestrahlung nur eine geringe Menge an dem codierten Protein detektiert werden. Gründe liegen vermutlich im starken Abbau der mRNA bei der chemischen Behandlung und der Schwierigkeit, die zahlreichen Photoschutzgruppen komplett abzuspalten [9].

Prof. Devaraj (University of California, San Diego) etablierte mit seinen Mitarbeitern einen chemoenzymatischen Ansatz (**Abb. 2B**). Dieser beruht auf einem Sekundärstrukturelement, das in die 5'-untranslatierte Region (UTR) der mRNA eingebracht wird. Die benötigte Stamm-Schleife-Struktur wird von der tRNA-Guanin-Transglycosylase (TGT) erkannt und ein Guanin wird normalerweise durch einen preQ1-Riboswitch ausgetauscht. Es gelang, ein synthetisches preQ1-Derivat mit einer Photoschutzgruppe herzustellen und die mRNA damit zu versehen (**Abb. 2B**). Insgesamt mussten drei Stamm-Schleife-Strukturen in die 5'-UTR eingebracht und mit je einer Photoschutzgruppe versehen werden, um die Translation in HeLa-Zellen drastisch zu reduzieren. Bestrahlung mit Licht von 405 Nanometern führte zu einer effizienten Translation der zuvor geschützten mRNA. Ein Nachteil ist lediglich, dass das Transkript mit einer permanent veränderten 5'-UTR versehen werden muss [10].

Prof. Ogasawara (Shinshu University, Matsumoto) entwickelte eine andere Strategie, mit der man die mRNA-Translation nicht nur an- sondern auch ausschalten kann. Dafür wurde die 5'-Kappe mit einem Photoschalter versehen, mit dem sich die Affinität von eIF4E zur 5'-Kappe modulieren lässt (**Abb. 2C**). Ist der Photoschalter in Trans-Konformation, wird die Translation größtenteils blockiert. In der Cis-Konformation wird die Translation hingegen nicht großartig gehindert. Mit diesem Ansatz gelang es sowohl in HeLa-Zellen als auch in Zebrafischen, die Translation an- und sogar wieder auszuschalten. Jedoch verbleibt die mRNA in allen Fällen chemisch modifiziert und kann sich im Organismus anders als eine native mRNA verhalten. Die relativ kurze Lebensdauer einer mRNA verringert

Hier steht
eine Anzeige.

Springer das Potenzial eines Photoschalters, da mehrere An-Aus-Zyklen kaum umsetzbar sind [11].

Unsere Arbeitsgruppe hat kürzliche eine neue Methode zur Lichtsteuerung der Translation entwickelt. Sie beruht auf einer synthetischen 5'-Kappe (Flash-Cap, **Abb. 3A**), die mit einer Photoschutzgruppe versehen ist und standardmäßig bei der *in vitro*-Transkription (IVT) eingesetzt werden kann, sodass man jede beliebige mRNA als Flash-Cap-mRNA herstellen kann (**Abb. 2D**). Die Einführung einer einzelnen Photoschutzgruppe an der 5'-Kappe blockiert die Bindung von eIF4E vollständig. Die kappenabhängige Translation ist auf wenige Prozent reduziert, kann aber durch kurze Bestrahlung mit Licht nahezu auf den Wert von mRNA mit einer regulären 5'-Kappe erhöht werden (**Abb. 3B**). Nach der Bestrahlung liegt die mRNA wieder komplett unmodifiziert vor und ist nicht von einer nativen mRNA unterscheidbar. Die Abspaltung der Photoschutzgruppe läuft schnell und effizient ab. Mithilfe der Flash-Cap-Technologie konnten wir verschiedene stummgeschaltete mRNAs in Säugerzelllinien einbringen und durch Bestrahlung die Translation aktivieren (**Abb. 3B**). Benutzt man zur Bestrahlung den Laser eines Konfokalmikroskops, kann die Translation auch lokal aktiviert werden (**Abb. 3C**, [12]).

Diese Methode ist somit ein nützliches Werkzeug, um die Effekte der ektopischen Genexpression mit räumlicher und zeitlicher Kontrolle in Zellen und Modellorganismen zu untersuchen. ■


Funding note: Open Access funding enabled and organized by Projekt DEAL.

Maria Dittmar 2016–2021 Chemiestudium an der Universität Münster. Seit 2021 Doktorarbeit in der Biochemie im Bereich der Nukleinsäurebiochemie.

Florian Peter Weissenböck 2014–2020 Chemiestudium an der Universität Münster. Seit 2020 Doktorarbeit in der Biochemie im Bereich der Nukleinsäurechemie.

Andrea Rentmeister 2002 Chemie-Diplom an der Universität Bonn. 2007 Promotion bei Prof. Dr. M. Famulok an der Universität Bonn im Bereich RNA-Biochemie. 2007–2010 Postdoktorat bei Prof. Dr. F. H. Arnold im Bereich Protein Engineering. 2010–2013 Juniorprofessur an der Universität Hamburg. Seit 2013 Professorin für Biologische Chemie und Biomolekulare Markierungschemie an der Universität Münster.


Literatur

[1] Zhang C Maruggi G Shan H Advances in mRNA Vaccines for Infectious Diseases Front Immunol 2019 10 594 10.3389/fimmu.2019.00594 30972078 PMC6446947
[2] Sahin U Karikó K Türeci Ö mRNA-based therapeutics — developing a new class of drugs Nat Rev Drug Discov 2014 13 759 780 10.1038/nrd4278 25233993
[3] Crick F Central Dogma of Molecular Biology Nature 1970 227 561 563 10.1038/227561a0 4913914
[4] Shirokikh N Preiss T Translation initiation by cap-dependent ribosome recruitment: Recent insights and open questions WIREs RNA 2018 9 e1473 10.1002/wrna.1473 29624880
[5] Mayer G Heckel A Biologically Active Molecules with a “Light Switch” Angew Chem Int Ed 2006 45 4900 4921 10.1002/anie.200600387 16826610
[6] Emiliani V Entcheva E Hedrich R Optogenetics for light control of biological systems Nat Rev Methods Primers 2022 2 55 10.1038/s43586-022-00136-4 PMC10627578 37933248
[7] Jeschik N Schneider T Meier C Photocaged and Mixed Photocaged Bioreversible-Protected ATP Derivatives as Tools for the Controlled Release of ATP Eur J Org Chem 2020 2020 6776 6789 10.1002/ejoc.202001229
[8] Darrah K Deiters A Translational control of gene function through optically regulated nucleic acids Chem Soc Rev 2021 50 13253 10.1039/D1CS00257K 34739027 PMC8900068
[9] Ando H Furuta T Tsien R Photo-mediated gene activation using caged RNA/DNA in zebrafish embryos Nat Genet 2001 28 317 325 10.1038/ng583 11479592
[10] Zhang D Zhou C Busby K Light-Activated Control of Translation by Enzymatic Covalent mRNA Labeling Angew Chem Int Ed 2018 57 2822 2826 10.1002/anie.201710917 PMC6052764 29380476
[11] Ogasawara S Duration Control of Protein Expressionin Vivoby Light-Mediated Reversible Activation of Translation ACS Chem Biol 2017 12 351 356 10.1021/acschembio.6b00684 28049292
[12] Klöcker N Weissenboeck F van Dülmen M Photocaged 5’ cap analogues for optical control of mRNA translation in cells Nat Chem 2022 14 905 913 10.1038/s41557-022-00972-7 35725774 PMC7613264
